# Closable Valves and Channels for Polymeric Microfluidic Devices

**DOI:** 10.3390/mi11070627

**Published:** 2020-06-27

**Authors:** Charles P. Clark, M. Shane Woolf, Sarah L. Karstens, Hannah M. Lewis, Aeren Q. Nauman, James P. Landers

**Affiliations:** 1 Department of Chemistry, University of Virginia, Charlottesville, VA 22903, USA; cpc4wg@virginia.edu (C.P.C.); msw2s@virginia.edu (M.S.W.); slk2ja@virginia.edu (S.L.K.); hml9wn@virginia.edu (H.M.L.); aqn6cd@virginia.edu (A.Q.N.); 2 Department of Electrical and Computer Engineering, University of Virginia, Charlottesville, VA 22903, USA; 3 Departments of Mechanical Engineering and Pathology, University of Virginia, Charlottesville, VA 22903, USA

**Keywords:** microfluidic, closable valving, centrifugal, expandable foam, redeposition, contact heating

## Abstract

This study explores three unique approaches for closing valves and channels within microfluidic systems, specifically multilayer, centrifugally driven polymeric devices. Precise control over the cessation of liquid movement is achieved through either the introduction of expanding polyurethane foam, the application of direct contact heating, or the redeposition of xerographic toner via chloroform solvation and evaporation. Each of these techniques modifies the substrate of the microdevice in a different way. All three are effective at closing a previously open fluidic pathway after a desired unit operation has taken place, i.e., sample metering, chemical reaction, or analytical measurement. Closing previously open valves and channels imparts stringent fluidic control—preventing backflow, maintaining pressurized chambers within the microdevice, and facilitating sample fractionation without cross-contamination. As such, a variety of microfluidic bioanalytical systems would benefit from the integration of these valving approaches.

## 1. Introduction

In the microscale regime, thoughtful integration of valving approaches permits directed fluid pumping, timed reagent release, mixing, metering, and the sealing of microfeatures. Single-shot, laser valves [1] offer a simple, easy to implement approach to valve opening. These destructible valves function well regardless of fluidic pressure or rotational frequency. Yet, they offer no resolution to the problem of microfeature sealing and closure, which is often required for accurately controlled fluidics. Microvalves that can be closed on-demand provide a way to seal chambers during heating or incubation steps allow for precise metering without backflow of reagents due to wicking and can prevent cross-contamination of reservoirs. Some current methods of closing microvalves are described hereafter.

Capillary [2,3,4,5], check, siphon [6,7,8], and hydrophobic [5,9,10,11] valves are well documented and play vital roles in microfluidic applications because they require few additional components or fabrication steps [12,13,14]. However, each of these passive valving tactics suffers from one or more operational limitations: (1) all are sensitive to perturbation and failure under high fluidic pressure (e.g., in centrifugal microfluidics, when high rotational frequencies are needed early in the analytical process), (2) most are limited to single-use actuation, and (3) with the exception of check valves, none are capable of sealing microfeatures to isolate liquid volumes or to prevent backflow. Conversely, phase-change ferro-wax [15,16] valves and elastomeric diaphragms, such as those described by Quake [17,18] and Mathies [19,20], provide true reversible valving capabilities.

Elastomeric diaphragms exploit the flexibility of polydimethylsiloxane (PDMS) membranes to selectively obstruct fluid flow. These valves are comprised of two primary layers—one fluidic and one pneumatic with a flexible, intervening membrane. Actuation and suppression of external pumps permits precise, complex, and dynamic microfluidic control. However, unavoidably, this approach requires substantial peripheral hardware and controls—driving up instrument size, complexity, and manufacturing costs.

Phase-change wax valves rely on the incorporation of meltable, paraffin-based ferrofluid to provide reversible valve operation [15]. Early examples of these valves required pneumatic/vacuum systems or integrated microheaters for full functionality. More recent iterations of this wax-based approach utilize laser irradiation to facilitate valve actuation, effectively supplanting microheaters, minimizing peripheral hardware, and simplifying operational control.

**Figure 1:**
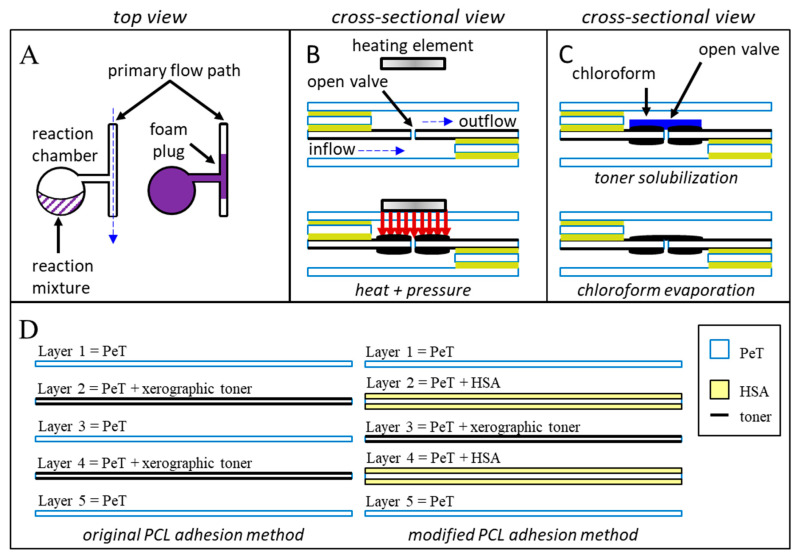
Schematic diagrams of the three putative closure methods. Method (**A**): Foam producing reagents are manually added to upstream reagent chambers within the device. Centrifugal pumping combines and mixes the two components. Upon mixing, the rapidly expanding polyurethane foam fills and blocks the target downstream channel. Method (**B**): Heat and pressure are applied directly to a previously opened laser hole. Controlled compression and melting of the polymeric layers and adhesives induce intermingling and permanent bonding of the polymeric materials. Upon cooling, a permanent, channel sealing weld is formed. Method (**C**): Chloroform is added to a previously opened laser patch, dissolving a portion of the xerographic toner. Rapid evaporation of the chloroform redeposits the toner into a uniform layer that covers the previously ablated laser hole. (**D**) Microdevices featured in this manuscript consisted of five laminated PeT films that were prepared and assembled using the “print-cut-laminate” (PCL) method of fabrication.

Herein, we propose three novel, alternative valve and channel closure methods. First, we demonstrate that rapidly expanding polyurethane foam creates physical barriers that can block channels to divert fluid flow. Polyurethane foam (FX Supply, A-B foam 2-lb density) is produced by combining a DABCO (1,4-diazabicyclo[2.2.2]octane) catalyst and a diisocyanate/diol mixture (Figure 1A) [21,22]. When mixed on-chip, these components react, forming carbon dioxide and a rapidly expanding polyurethane plug which, upon hardening, obstructs previously open microfeatures. Thoughtful microchip design is required to store reagents separately prior to desired expansion and channel blockage.

Second, we explore the pinpoint application of heat and pressure as a means for creating physical barriers between previously connected microfluidic chambers (Figure 1B). Much like the spot-welding techniques used in automotive manufacturing or the heat-sealing methods used in the food industry, our approach uses a heated, rod-like pin to melt and irreversibly join plastic monolayers. The schematic depicted in Figure 1B shows the application of heat over a microchannel and fluidic via; under heat and pressure, polymeric the underlying plastic films will rapidly soften, melt, intermingle, and (upon cooling) reharden to form permanent welds.

Finally, we characterize a truly multiuse valve by treating a xerographic toner patch (2 mm × 2 mm) with chloroform. Previously published work describes the use of xerographic toner, or other optically dense materials, to absorb intense laser light, facilitating valve opening [1]. We hypothesized that a portion of the toner could be repurposed to seal previously opened laser holes by way of chloroform dissolution and redeposition (Figure 1C). Rather than a one-time blockage of an otherwise open microchannel, this chloroform redeposition approach seeks to produce a valve that can be repeatedly opened and closed as desired.

## 2. Materials and Methods

### 2.1. Microchip Fabrication

All microdevices described in this manuscript were fabricated using the “print-cut-laminate” (PCL) method [11,23]. The ability to swiftly manufacture chips combined with leveraging low-cost materials makes this fabrication approach ideal for rapid prototyping and methods development. With the exception of microchips for testing the expanding foam method, each microfluidic device consisted of five laminated polyethylene terephthalate (PeT) films (Film Source, Inc. Maryland Heights, MO, USA) with intervening heat-sensitive adhesive (HSA) layers (EL-7970-39, Adhesives Research, Inc. Glen Rock, PA, USA) (Figure 1D, left). The expanding foam approach used toner-printed layers of PeT, rather than HSA, to provide layer adhesion (Figure 1D, right). Microfluidic architectures were ablated into the polymeric layers with a CO_2_ laser (VLS3.50, Universal^®^ Laser Systems, Scottsdale, AZ, USA). An office laminator was used for the final heat-pressure bonding step (UltraLam 250B, Akiles Products, Inc., Mira Loma, CA, USA). Generally, all pneumatic vents and inlets were ablated into layer 1, while all chambers were ablated into layers 2–4. Layers two and four served as the primary fluidic via layers, encompassing all inflow and outflow channels. Layer three provided laser-actuated valve opening capabilities (Figure 1B,C). Two coats of black xerographic toner were applied to each side of those middle valving layers (Figure 1D) (LaserJet 4000, Hewlett-Packard, Palo Alto, CA, USA).

### 2.2. Valve Opening

Cordero el al. described a simple, laser-based method for valve opening [1]. For the contact heating and chloroform redeposition studies, valves were opened by irradiating each xerographic toner patch (2 mm × 2 mm) with a 700 mW 638 nm laser diode (L638P700M, Thorlabs, Inc., Newton, NJ, USA). Toner patches were irradiated at 500 mW for 0.5 s from a z-height of 15 mm. Irradiation under these conditions fashioned holes that were 80 to 100 µm diameter (Ø).

### 2.3. Experimental Conditions

#### 2.3.1. Expanding Foam

**Figure 2:**
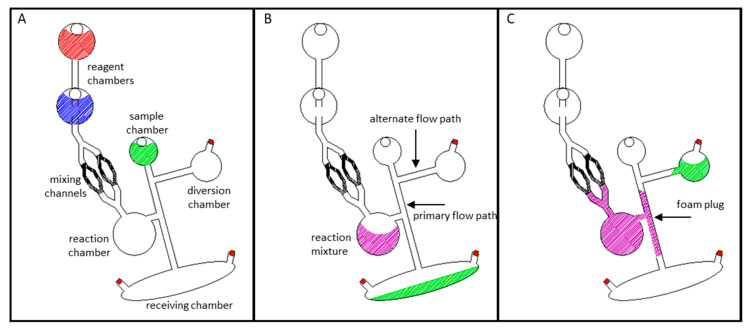
Blocking a channel with expanding polyurethane foam. (**A**) Schematic of the microchip prior to testing. The two reagents are separated, with architecture to mix them before sample is diverted. (**B**) Prior to channel blockage, dye solution freely flows through the open channel architecture. (**C**) Upon foam expansion and curing, the primary channel (2 mm diameter) is blocked. When new sample is added and the microdevice spun, fluid is diverted into the previously unfavorable flow path.

To test the expanding foam approach for channel occlusion, a microchip comprised of a sample reservoir, two downstream collection reservoirs, and a reaction chamber was constructed (Figure 2A and Supplementary Figure S1). To demonstrate that aqueous fluids flow freely through this architecture, green dye was added to the sample reservoir and centrifugally pumped into the large receiving chamber nearer the periphery of the disc (1000 rpm for 30 s) [12,13,24]. An illustration of this is depicted in Figure 2A,B. To establish the ability of the polyurethane foam to halt subsequent fluid flow, reagent parts A and B (5 µl each) were loaded into separate reservoirs in the microchip (Figure 2A—red and blue reagent chambers) and then centrifugally driven into the reaction chamber (Figure 2B—purple reaction mixture). Mixing produced polyurethane foam that expanded into, and solidified within, the adjacent microfluidic channel (primary flow path), creating a rigid plug (illustrated in purple in Figure 2C). Much like gases expanding to fill a container, the increasing volume of the reacting foam solution is constrained by the microfluidic architecture. Thus, the foam expands to fill the available space, i.e., the reaction chamber and the adjacent microchannel (Figure 2C—purple). Thoughtful architectural design and tailoring of the reagent volumes minimized foam expansion beyond the upstream mixing channels and permitted the preferential filling of the primary flow path. Explicitly, (1) the primary flow path is positioned closer to the bottom of the reaction/expansion chamber and is thus encountered first. (2) The upstream mixing channels exhibit a greater resistance to flow (and foam expansion) due to the narrower diameter and smaller cross-sectional area relative to the primary flow path. (3) If necessary, the expanding foam front can be forced peripherally (away from the center of rotation and away from the upstream channels) by maintaining device rotation. We have, however, found this later consideration to be largely unnecessary if appropriate volumes of the reactants are utilized. Foam curing time ranged from 8 to 14 min (*n* = 4 each in 1 min increments). To evaluate and challenge the integrity of foam channel closures, green dye was added to the sample reservoir and centrifugally pumped for 30 s (1000 rpm). When challenged, successful foam closures would completely occlude the target microfluidic channel, effectively preventing fluid flow between the sample chamber and the receiving chamber. Essentially, fluid would be rerouted into a secondary diversion chamber (Figure 2C).

#### 2.3.2. Contact Heating

**Figure 3:**
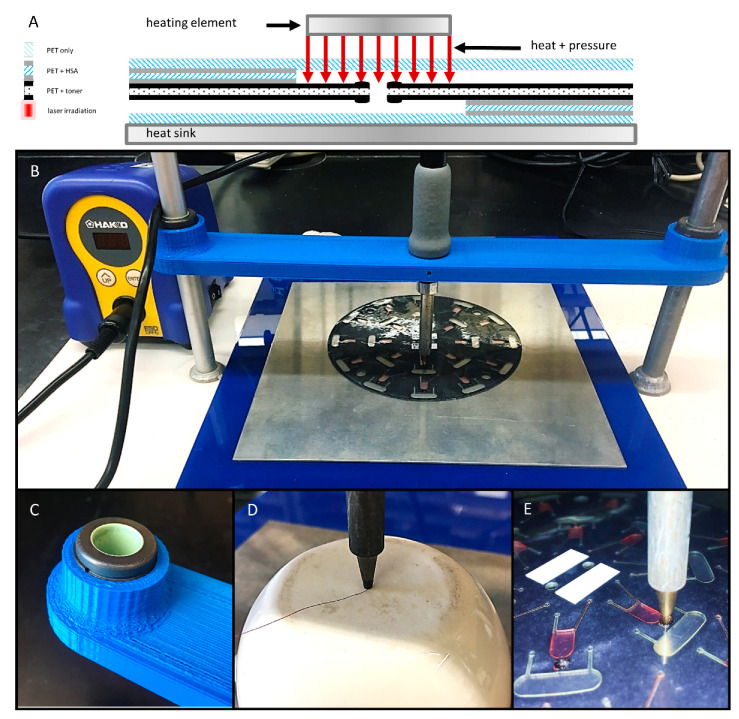
Schematic diagram and photographs of the gantry platform for bringing the heating element into contact with the disc. (**A**) Schematic diagram of the contact heating approach. (**B**) A gantry arm was 3D printed, outfitted with two self-aligning linear sleeve bearings (**C**), and suspended between two standard laboratory support stands. The soldering stylus was inserted into the gantry arm, held in place with set screws, and connected to the adjustable power supply. (**D**) Calibration of stylus temperature with a digital thermometer and a T-type thermocouple. (**E**) Close-up photograph of the soldering stylus in contact with a previously opened laser valve (2 × 2 mm).

Preliminary evaluation of this closure method involved a sample set of 181 valves that were previously opened via laser irradiation. To confirm valve opening, water (10 µL) was loaded into a chamber nearer the center of rotation, centrifugally driven through the open valve (1000 rpm for 3 s), and collected in a downstream receiving chamber. For these preliminary experiments, heat and pressure were manually applied to previously opened laser valve with a soldering stylus (FX-8801, HAKKO Corp. Naniwa-ku, Osaka, Japan) with an adjustable 70 W power supply (FX-888D-23BY, Hakko) (Figure 3A). The conical stylus tip (T18-B, Hakko) was modified such that the end was flat with a diameter ø = 1.5 mm. Stylus temperature ranged from 100 °C to 180 °C. Stylus temperature was calibrated and verified with a Fluke 54 II B 60 HZ dual input thermometer and a T-type thermocouple (Fluke Corp., Everett, WA, USA). A 1.5 mm thick 6061 aluminum alloy plate served as a heat sink. Contact time and applied pressure varied from 10–30 s and 110–965 psi, respectively. (Figure 3D). During this preliminary evaluation, all valve closures were challenged by loading 10 µl of colored dye into the upstream chamber and exposing to a single 5 s spin at 2000 rpm (approximately 245 *g). Successful valve closures were defined as a closure that remained leak-free with no apparent fluid seepage or outflow.

To better understand the effects of and the relationship between the three key predictor variables (time, temperature, and pressure), a second series of experiments was conducted. Briefly, to ensure uniform contact with the 2 mm × 2 mm laser patches, a custom gantry arm was 3D printed, outfitted with two, self-aligning linear sleeve bearings (McMaster-Carr, Elmhurst, IL, USA) and suspended between two standard laboratory ring stands (Figure 3B,C). The soldering stylus was inserted into the gantry arm and held in place with two set screws. The same adjustable 70 W power supply (FX-888D-23BY, Hakko) and modified conical stylus tip (T18-B, Hakko) were utilized. This gantry arm setup was used to evaluate an additional 180 valve closures (10 discs, *n* = 18 valves each). As before, water was passed through the previously opened valves to ensure successful opening. Valves closures were attempted at 18 different temperatures ranging from 100 °C to 270 °C (*n* = 10 each). Contact time and applied pressure were held constant at 3 s and 336 ± 75 psi, respectively.

During this second set of experiments, all valve closures were challenged by loading 10 µL of dye into the upstream chamber (Figure 3E) and exposing to three sequential 5 s spins at 2000, 3000, and 4000 rpm (approximately 245, 555, and 985 *g, where *g is the conventional value of gravitational acceleration at the Earth’s surface). Successful valve closures were defined as a closure that remained leak-free through the 985 *g spin with no apparent fluid loss or escape. To be clear, slow or partial leaks were recorded as complete failures at the earliest indication of dye ingress into the downstream chamber and/or fluid egress from the loading chamber. Thus, a valve closure was deemed successful only if the weld remained leak-free through the 985 *g spin.

#### 2.3.3. Chloroform Redeposition of Toner

To demonstrate that redeposited toner can withstand centrifugally generated hydraulic pressure, we sequentially released and isolated four different-colored dyes by selectively opening and closing downstream valves via laser-actuation and subsequent chloroform redeposition. For this experiment, each chip consisted of four sample reservoirs, a single centralized “mixing” chamber and channel, with four adjoining collection reservoirs. First, two laser valves were opened to release a single dye sample-one beneath the dye loading reservoir and one leading to the target collection reservoir. Rotationally generated forces propelled the dye solution through the mixing chamber and into the collection reservoir (1000 rpm 25 s counterclockwise and clockwise. Prior to the release and centrifugal pumping of the next dye solution, the previously opened, lower laser patch was closed. Each valve was treated with 1.5 µL chloroform (Fisher Scientific, Waltham, MA, USA, 99.9%). Explicitly, chloroform was pipetted onto each toner patch via an air vent and allowed to evaporate at room temperature for 120 s. This sequence was repeated for each of the remaining dye samples.

## 3. Results & Discussion

### 3.1. Expanding Foam Method

**Figure 4:**
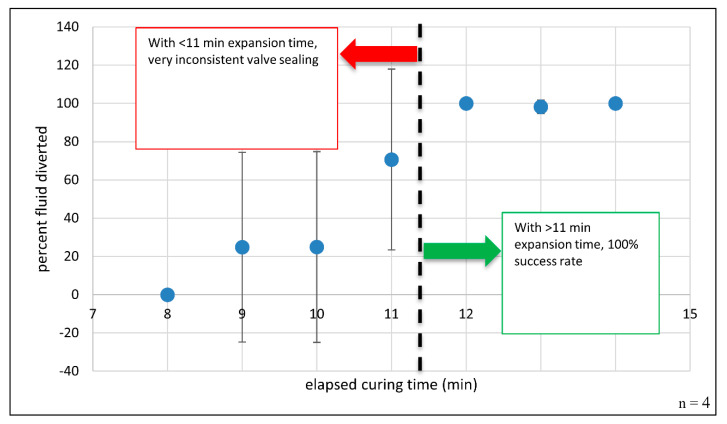
Blocking a channel with expanding polyurethane foam: Percent fluid diversion as a function of elapsed time. When the foam mixture was allowed to expand and cure for ≥12 min, 100% fluid diversion was observed. *n* = 4 attempted closures per time point. Note: When curing time > 11 min, the error bars are quite small and are not readily visible at 12- and 14-min time points.

By and large, we chose to explore expanding foam as a valving option due to the physical properties of the foam and the simplicity of the application, i.e., does not require external heating or mechanical manipulation for activation. Briefly, upon activation, the foam mixture expands 30–60 times, generating a rigid mass that forms an airtight, waterproof seal. Thus, filling, conforming to, and sealing a microchannel would require very small reagent volumes. However, one unknown factor was the minimum expansion and curing time required for complete channel sealing to occur. JPEG images (24-bit color 96 dpi) of each tested microdevice were acquired with a desktop scanner (Perfection V100 Photo, Epson, Suwa, Nagano Prefecture, Japan). Sections of the images containing the diversion and receiving chambers were selected, cropped, and analyzed within ImageJ software [25,26,27]. Green pixels were identified and selected using the ImageJ *Adjustment* and *Color Threshold* modules. Explicitly, green pixels were isolated and selected by applying upper and lower thresholds for the hue (50 to 255), saturation (100 to 255), and brightness channels (70 to 255). To quantify the total green pixel area in each chamber, the ImageJ *Measure* command was used to evaluate each selected region. Percent dye diversion was used as a metric for assessing foam valve closure success, which was calculated as

(1) $$\%\;diversion=100(A_{div}/\;A_{tot}),$$

where $A_{div}$ is the green pixel area in the diversion chamber, and $A_{tot}$ is the sum of the selected green pixel areas within the receiving and diversion chambers. This process is detailed in Supplementary Figure S2. The results of testing polyurethane foam-based channel blockage are shown in Figure 4. The percent fluid diverted is plotted against elapsed foam expansion time. Error bars for this plot represent ± one sample standard deviation. Error bars for curing time points ≤ 11 min are quite large, suggesting significant variability in the results and indicating a high degree of error or uncertainty in the reported means, i.e., how far the error-free, true mean might be from the reported value. Negative error bar values at time points 9- and 10-min should not be taken to suggest that fluid diversion is somehow negative or that reverse fluid pumping occurs. From this plot, it is apparent that channel blockage is inconsistent and leads to failed fluid diversion when dye is added and centrifugally pumped prior to 12 min of total curing time. However, this plot also clearly shows reproducible channel occlusion when the foam is left to cure for 12 min or more. With thoughtful architectural design, careful tailoring of the reaction components, and adequate curing time, the resulting foam plug completely seals the channel with comprehensive fluid diversion into the previously unfavorable fluidic path, suggesting that individual microfeatures can be selectively and completely obstructed in as little as 12 min. This prolonged curing step could be performed in parallel with a variety of common biochemical and analytical processes, like thermal cycling during DNA amplification via polymerase chain reaction (PCR), chemical/enzymatic cellular lysis, and magnetic mixing for bead-based dynamic solid phase extraction [28]. Foam-forming regents can be deposited via simple, on-disc reagent storage methods, such as blister pouches or custom ampules [29,30]. By avoiding the need for manual pipetting and user intervention, reagent-containing pouches and ampules provide a path to timed reagent release and the complete, automated integration of this valving approach.

### 3.2. Contact Heating Method

**Figure 5:**
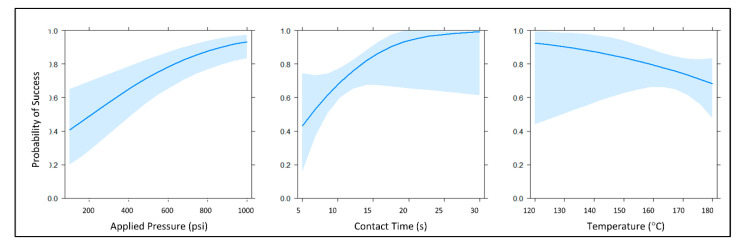
Main effect plots of the selected generalized linear model. The main effects for this model were the continuous predictor variables applied pressure, temperature, and contact time (power + height + time). Each of these marginal effects plots reflects the probability of successful channel closure when the other predictor variables are held constant at their respective means. Light blue traces indicate a 95% confidence interval for each probability plot.

By investigating contact heating as a means of channel closure, we sought to ameliorate two potential concerns associated with the expandable foam and chloroform redeposition methods, namely to eliminate the need for any additional reagents and to circumvent the need for prolonged curing and evaporation. Logistic regression is a popular and effective way of modeling binary response data (i.e., success-failure, yes-no, 0–1, etc.). Logistic regression yields a mathematical model that can be used to estimate the probability of valve or channel closure given certain independent variables (e.g., pressure, time, and temperature) and their interactions. The logistic function used in these models estimates probabilities and produces coefficients that are on the log-odds or logit scale, essentially a list of numbers that can be difficult to translate into an intuitive, useful form [31]. However, graphical displays, provide an intuitive, visual means of understanding these complex mathematical models. For the preliminary contact heating experiment, eight candidate logistic regression models were created using three key predictor variables: pressure, time, and temperature (main effects) (Figure 5). These logistic regression models were fashioned using the glm function in R version 3.6.1 (05 July 2019) and were selected using the Akaike information criterion (AIC) [32]. Models with lower AIC values were considered better fits for these data. AIC differences were calculated as

(2) $$\Delta AIC_{i}=AIC_{i}-AIC_{\min},$$

where $AIC_{i}$ is the AIC for the ith model, and $AIC_{\min}$ is the AIC of the lowest scoring (minimum) model. ANOVA was used to establish statistical significance for each main effect and the interaction terms [33]. Marginal effects plots were generated using the *effects* package in R [34,35,36]. For the second experiment, when contact time and applied pressure were held constant, temperature served as the sole predictor variable, and a single candidate regression model was created.

The highest quality statistical model was determined via the Bayesian information criterion (BIC) and Akaike information criterion (AIC) evaluations. The core summary() command in R returned z-statistics, as well as the concomitant *p*-values for each main effect term in the model, suggesting that all three predictor variables were statistically significant at α = 0.05 (*z* = 2.218, 1.999, 3.838; and *p* = 0.026585, 0.045645, 0.000124 for temperature, contact time, and applied pressure, respectively). Generally speaking, data analysis of this initial experiment suggested that increasing contact time and pressure (applied force) had positive effects on the probability of successful valve closure, while increasing temperature exhibited a negative effect on the probability of successful valve closure. When testing the null hypothesis that there is no interaction between stylus temperature and contact time, we reject the null hypothesis at α = 0.05. Stated another way, this modeling indicated a significant two-way interaction between temperature and time, although this interaction was not overwhelmingly significant at α = 0.05 (*z* = −2.004, *p* = 0.045039). Collectively, these preliminary findings indicated that it is possible to close previously opened laser vales at lower temperatures. However, increased pressure and heating time were required. Thus, we surmised that the apparent negative effect of temperature on the probability of successful valve closure was due, at least in part, to the aforementioned complex two-way interaction between stylus temperature and contact time. As such, we hypothesized that evaluating stylus temperature as the sole predictor variable, while contact time and applied pressure were held constant, would reveal more optimal closure conditions with a smaller, more favorable confidence interval in the probability of successful closure.

**Figure 6:**
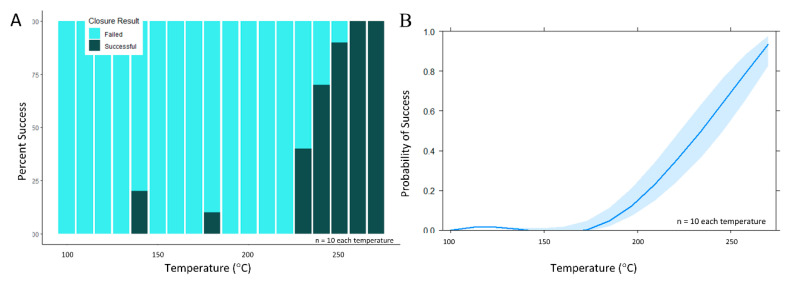
Evaluation of valve closure via the contact heating method over a wider temperature range. (**A**) When pressure and time were held constant (336 ± 75 psi and 3 s, respectively) and as stylus temperature exceeded 230 °C, a sharp increase in channel closure success was noted. (**B**) Broadly speaking, this effects plot visualization of the generalized linear model suggests a rapid increase in the probability of successful closure as stylus temperature increases from 200 to 270 °C.

During the secondary set of experiments, pressure and contact time were held constant while temperature varied. A sharp increase in the number of successful channel closures was noted as stylus temperature exceeded 230 °C approached the melt temperature of PeT (*T_M_* = 250 °C) (Figure 6A,B). Specifically, when stylus temperature was 250 °C, 93.2% of valve closures (*n* = 73) remained leak-free to 4000 rpm (982.3 *g). This finding is significant given that most spin systems operate at modest rotational frequencies of 600–3000 RPM (10–50 Hz) [6,12,13,15,37,38,39] and that most centrifugal microfluidic discs are no larger than a standard compact disc (120 mm ⌀), i.e., most valves will experience a relative centrifugal force (rcf) ≤ 605 *g. Using temperature as the sole predictor variable for this data set, a single candidate generalized linear model was created. Statistical modeling of these data confirmed that temperature was a statistically significant main effect term at α = 0.05 (*p* = 6.12 × 10^−10^).

Contact heating offers a straightforward approach to microfeature closure that is flexible and customizable, allowing the user to tune applied pressure, as well as heating element temperature and dwell time. This strategy for closing microfeatures is a striking alternative to other active valve closure methods (e.g., elastomeric diaphragms, ferrowax systems, polyurethane foam, etc.) for two key reasons. First, no additional waxes, foaming agents, solvents, or other reagents are required. Second, and perhaps most apropos, this versatile approach acts on existing microfeatures and requires no additional fabrication steps, changes to microfluidic architecture, nor supplementary chemical reagents. From an engineering perspective, integration of the requisite heating element is rather simple. That is, this contact heating approach requires little more than a retractable heating element. Alternatively, its is conceivable that a microheater could be applied locally to the device and/or valve, effectivley minimizing engineering complexity by elminating the need for a retractable heating element.

### 3.3. Chloroform Redeposition Method

**Figure 7:**
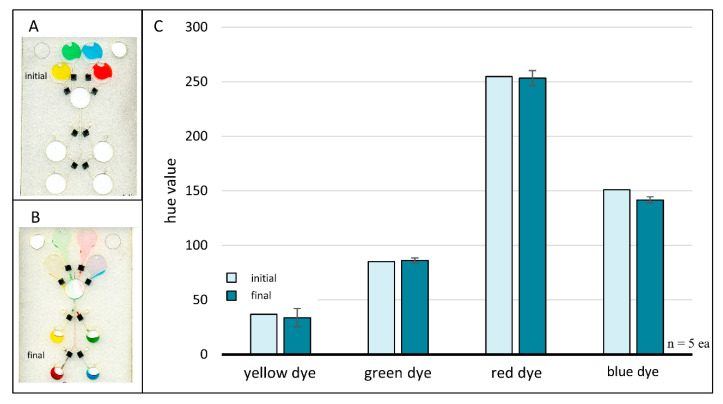
Chloroform redeposition of toner. A loaded four-domain chip prior to any fluid flow or valving events (**A**) and after following completion of all fluid flow and valving events (**B**). Valves were sealed using chloroform for dissolution and redeposition of xerographic toner patches. Scanned images were acquired with an Epson Perfection V100 Photo desktop scanner (Japan). Bar plot (**C**) comparing initial and final hue values for each colored dye. Differences in initial and final hue values were negligible, suggesting successful valve closures via chloroform redeposition of xerographic toner.

Due to the relatively small hole generated by laser ablation during valve opening, we believed that chemical lifting and redeposition of toner ink would be sufficient for diverting subsequent liquid flow. Relative to the total size of the toner patch (2 mm × 2 mm) the laser ablated hole is quite small (approximately 80 µm diameter). Chloroform was used to solubilize some of the xerographic toner ink surrounding the newly opened laser valves. Those dissolved toner particles were redistributed and redeposited at the liquid-solid interface during chloroform evaporation, effectively covering the laser ablated hole. Thus, with minimal changes to the microfluidic architecture, we targeted chloroform redeposition as a potential candidate for true multiuse valving, i.e., one that could be opened and then re-closed at will. Initial and final JPEG images (24-bit color 96 dpi) were captured with a desktop scanner (Epson, Japan) (Figure 7A,B). Sections of each image were selected, cropped, and individually analyzed within ImageJ software [25,26,27]. Specifically, sectors containing the loading chamber were cropped from the initial images, while regions containing the receiving chambers were cropped from the final images. Cropped regions were converted to 3-slice HSB stacks (Hue, Saturation, Brightness) via the ImageJ *Image > Type* submenu. Mean hue values for each chamber were obtained with the ImageJ *Measure* command. Valve closure was evaluated by comparing the initial hue value of the loading chamber with the final hue value of the corresponding receiving chamber. Any deviation from the initial hue values indicated that at least one valve failed to completely prevent fluid flow after being closed via chloroform redeposition of toner. To prevent unintentional biases towards preferential valve performance, experiments were conducted by spinning in both rotational directions, as well as changing the order in which valves were closed.

Image analysis results for the four-domain chloroform redeposition microchip studies are shown in Figure 7C. Control data points (n*_initial_* = 1 each) represent the initial hue value of each colored dye when added directly to the chip, i.e., prior to any fluid movement or valving events. Sample data points represent the final hue value of each dye volume and are an average of five trials (*n_final_* = 5 each) with error bars representing one standard deviation. This plot plainly shows no appreciable hue shifts, suggesting that chloroform redeposition of toner is a viable method for rapid closure of previously opened laser holes.

We are aware that chloroform is incompatible with some biological assays, especially those involving downstream DNA amplification [40]. However, thoughtful architectural design to allow for adequate evaporation or venting may prove sufficient to prevent chloroform interaction with the biological sample. Moreover, the other valving options described here provide viable alternatives and may be more well-suited for use in some biochemical assays. Nevertheless, we have demonstrated that chloroform redeposition of toner is an adequate method for sealing valves after laser-actuated opening and sample flowthrough.

Little to no added architectural complexity, small reagent volumes, and rapid evaporation time (minimal workflow disruption) make this this solvent-based redeposition approach an attractive valve closure option. Chloroform can be deposited via simple, on-disc reagent storage methods, such as blister pouches or custom ampules [29,30]. By avoiding the need for manual pipetting and user intervention, chloroform containing pouches and ampules provide a path to timed reagent release and the complete, automated integration of this valving approach.

## 4. Conclusions

We successfully demonstrated proof-of-concept for three novel approaches to sealing valves and channels within of microfluidic devices, each with distinct advantages and considerations. (Table 1) It should be noted that of the three putative closure methods, chloroform is the only truly reversible/reusable approach. As reported in Table 1, contact heating was easily the fast and strongest of the three methods. Although the expanding foam and chloroform closures were only evaluated at modest rotational frequencies with modest hydraulic pressure heads (i.e., 1000 rpm = 67 *g), we suspect that, when challenged, foam closures will be stronger than reported herein. While the scope of this article is limited to PCL fabricated polymeric, centrifugal microfluidic devices consisting largely of PeT, xerographic toner, and HSA, we suspect that these methods will prove useful on a broad range of microdevices that contain or are comprised of other polymeric materials (e.g., poly(methyl methacrylate) (PMMA), PDMS, cyclic olefin copolymer (COC), etc.) [41]. By avoiding the need for external pneumatic pumping, nanoheaters, and laser diodes, these newly described procedures complement existing active valve opening and fabrication techniques by offering versatile, relatively inexpensive alternatives to elastomeric diaphragms and phase-change wax systems. Thus, effectively expanding the valving options in the existing microfluidic toolbox.

## Figures and Tables

**Table 1:** Potential considerations for implementation of the three putative closure methods. Valve strength was evaluated by applying hydraulic pressure to attempted valve and channel closures.

	Architectural Complexity	Additional Reagents	Prolonged Curing	Engineering Considerations	Total Time required	Reversibility or Reusability	Closure Strength
**Expanding Foam**	**×**	**×**	**×**		>11 min	no	≥60 *g
**Chloroform**		**×**			120 s	yes	≥60 *g
**Contact Heat**				**×**	3 s	no	≥985 *g

## Supplementary Materials

The following are available online at https://www.mdpi.com/2072-666X/11/7/627/s1, Figure S1: Architectural schematic of the chloroform redeposition device, Figure S2: Quantitative analysis of diverted fluid.

## References

[1] Garcia-Cordero J.L., Kurzbuch D., Benito-Lopez F., Diamond D., Lee L.P., Ricco A.J. (2010). Optically addressable single-use microfluidic valves by laser printer lithography. Lab Chip.

[2] Zhang C., Xing D., Li Y. (2007). Micropumps, microvalves, and micromixers within PCR microfluidic chips: Advances and trends. Biotechnol. Adv..

[3] Glière A., Delattre C. (2006). Modeling and fabrication of capillary stop valves for planar microfluidic systems. Sens. Actuators A Phys..

[4] Maria M.S., Rakesh P., Chandra T., Sen A. (2017). Capillary flow-driven microfluidic device with wettability gradient and sedimentation effects for blood plasma separation. Sci. Rep..

[5] Tsougeni K., Papageorgiou D., Tserepi A., Gogolides E. (2010). “Smart” polymeric microfluidics fabricated by plasma processing: Controlled wetting, capillary filling and hydrophobic valving. Lab Chip.

[6] Siegrist J., Gorkin R., Clime L., Roy E., Peytavi R., Kido H., Bergeron M., Veres T., Madou M. (2010). Serial siphon valving for centrifugal microfluidic platforms. Microfluid. Nanofluidics.

[7] Steigert J., Brenner T., Grumann M., Riegger L., Lutz S., Zengerle R., Ducrée J. (2007). Integrated siphon-based metering and sedimentation of whole blood on a hydrophilic lab-on-a-disk. Biomed. Microdevices.

[8] Gorkin R., Soroori S., Southard W., Clime L., Veres T., Kido H., Kulinsky L., Madou M. (2012). Suction-enhanced siphon valves for centrifugal microfluidic platforms. Microfluid. Nanofluidics.

[9] Feng Y., Zhou Z., Ye X., Xiong J. (2003). Passive valves based on hydrophobic microfluidics. Sens. Actuators A Phys..

[10] Ouyang Y., Wang S., Li J., Riehl P.S., Begley M., Landers J.P. (2013). Rapid patterning of ‘tunable’ hydrophobic valves on disposable microchips by laser printer lithography. Lab Chip.

[11] Thompson B.L., Ouyang Y., Duarte G.R., Carrilho E., Krauss S.T., Landers J.P. (2015). Inexpensive, rapid prototyping of microfluidic devices using overhead transparencies and a laser print, cut and laminate fabrication method. Nat. Protoc..

[12] Gorkin R., Park J., Siegrist J., Amasia M., Lee B.S., Park J.-M., Kim J., Kim H., Madou M., Cho Y.-K. (2010). Centrifugal microfluidics for biomedical applications. Lab Chip.

[13] Ducrée J., Haeberle S., Lutz S., Pausch S., Von Stetten F., Zengerle R. (2007). The centrifugal microfluidic Bio-Disk platform. J. Micromechanics Microeng..

[14] Mark D., Haeberle S., Roth G., Von Stetten F., Zengerle R. (2010). Microfluidic lab-on-a-chip platforms: Requirements, characteristics and applications. Microfluidics Based Microsystems.

[15] Park J.-M., Cho Y.-K., Lee B.-S., Lee J.-G., Ko C. (2007). Multifunctional microvalves control by optical illumination on nanoheaters and its application in centrifugal microfluidic devices. Lab Chip.

[16] Oh K.W. (2005). A phase change microvalve using a meltable magnetic material: Ferro-Wax. Bio Lab Samsung Adv. Inst. Technol..

[17] Thorsen T., Maerkl S.J., Quake S.R. (2002). Microfluidic large-scale integration. Science.

[18] Melin J., Quake S.R. (2007). Microfluidic large-scale integration: The evolution of design rules for biological automation. Annu. Rev. Biophys. Biomol. Struct..

[19] Grover W.H., Ivester R.H., Jensen E.C., Mathies R.A. (2006). Development and multiplexed control of latching pneumatic valves using microfluidic logical structures. Lab Chip.

[20] Zhang W., Lin S., Wang C., Hu J., Li C., Zhuang Z., Zhou Y., Mathies R.A., Yang C.J. (2009). PMMA/PDMS valves and pumps for disposable microfluidics. Lab Chip.

[21] Gama N.V., Ferreira A., Barros-Timmons A. (2018). Polyurethane foams: Past, present, and future. Materials.

[22] Chattopadhyay D., Webster D.C. (2009). Thermal stability and flame retardancy of polyurethanes. Prog. Polym. Sci..

[23] Birch C., DuVall J.A., Le Roux D., Thompson B.L., Tsuei A.-C., Li J., Nelson D.A., Mills D.L., Landers J.P., Root B.E. (2017). Rapid Fabrication of Electrophoretic Microfluidic Devices from Polyester, Adhesives and Gold Leaf. Micromachines (Basel).

[24] Strohmeier O., Keller M., Schwemmer F., Zehnle S., Mark D., von Stetten F., Zengerle R., Paust N. (2015). Centrifugal microfluidic platforms: Advanced unit operations and applications. Chem. Soc. Rev..

[25] Rueden C.T., Schindelin J., Hiner M.C., DeZonia B.E., Walter A.E., Arena E.T., Eliceiri K.W. (2017). ImageJ2: ImageJ for the next generation of scientific image data. BMC Bioinform..

[26] Schneider C.A., Rasband W.S., Eliceiri K.W. (2012). NIH Image to ImageJ: 25 years of image analysis. Nat. Methods.

[27] Schindelin J., Arganda-Carreras I., Frise E., Kaynig V., Longair M., Pietzsch T., Preibisch S., Rueden C., Saalfeld S., Schmid B. (2012). Fiji: An open-source platform for biological-image analysis. Nat. Methods.

[28] Jackson K., Borba J., Meija M., Mills D., Haverstick D., Olson K., Aranda R., Garner G., Carrilho E., Landers J. (2016). DNA purification using dynamic solid-phase extraction on a rotationally-driven polyethylene-terephthalate microdevice. Anal. Chim. Acta.

[29] Smith S., Sewart R., Becker H., Roux P., Land K. (2016). Blister pouches for effective reagent storage on microfluidic chips for blood cell counting. Microfluid. Nanofluidics.

[30] Krauss S.T., Woolf M.S., Hadley K.C., Collins N.M., Nauman A.Q., Landers J.P. (2019). Centrifugal microfluidic devices using low-volume reagent storage and inward fluid displacement for presumptive drug detection. Sens. Actuators B Chem..

[31] Harrell F.E. (2015). Regression Modeling Strategies: With Applications to Linear Models, Logistic and Ordinal regression, and Survival Analysis.

[32] Sakamoto Y., Ishiguro M., Kitagawa G. (1986). Akaike Information Criterion Statistics.

[33] Dziak J.J., Coffman D.L., Lanza S.T., Li R., Jermiin L.S. (2019). Sensitivity and specificity of information criteria. bioRxiv.

[34] Fox J., Weisberg S. (2019). An R Companion to Applied Regression.

[35] Fox J., Weisberg S. (2018). Visualizing Fit and Lack of Fit in Complex Regression Models with Predictor Effect Plots and Partial Residuals. J. Stat. Softw..

[36] Fox J., Weisberg S. (2003). Effect Displays in R for Generalised Linear Models. J. Stat. Softw..

[37] Duffy D.C., Gillis H.L., Lin J., Sheppard N.F., Kellogg G.J. (1999). Microfabricated Centrifugal Microfluidic Systems:  Characterization and Multiple Enzymatic Assays. Anal. Chem..

[38] Mark D., Weber P., Lutz S., Focke M., Zengerle R., von Stetten F. (2011). Aliquoting on the centrifugal microfluidic platform based on centrifugo-pneumatic valves. Microfluid. Nanofluidics.

[39] Mark D., Metz T., Haeberle S., Lutz S., Ducrée J., Zengerle R., von Stetten F. (2009). Centrifugo-pneumatic valve for metering of highly wetting liquids on centrifugal microfluidic platforms. Lab Chip.

[40] Rossen L., Nørskov P., Holmstrøm K., Rasmussen O.F. (1992). Inhibition of PCR by components of food samples, microbial diagnostic assays and DNA-extraction solutions. Int. J. Food Microbiol..

[41] Tsao C.-W. (2016). Polymer microfluidics: Simple, low-cost fabrication process bridging academic lab research to commercialized production. Micromachines (Basel).

