# Supplementary figures and images for: Closable Valves and Channels for Polymeric Microfluidic Devices
Source: Micromachines (Basel). 2020 Jun 27;11(7):627. doi: 10.3390/mi11070627 (PMC7407107; doi:10.3390/mi11070627)

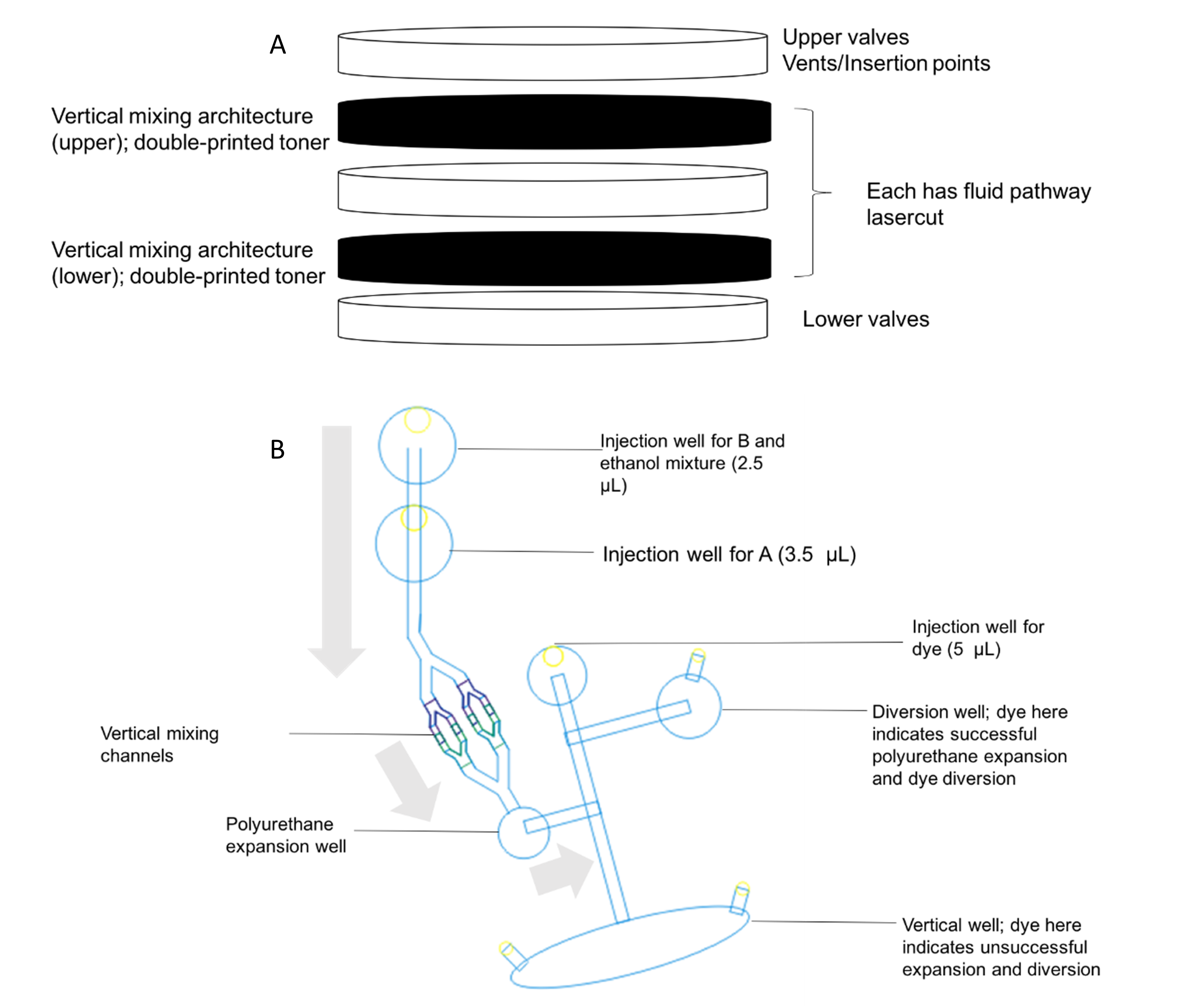

Supplement: Supplementary file 1 [file micromachines-11-00627-s001.zip › Slide1.TIF]

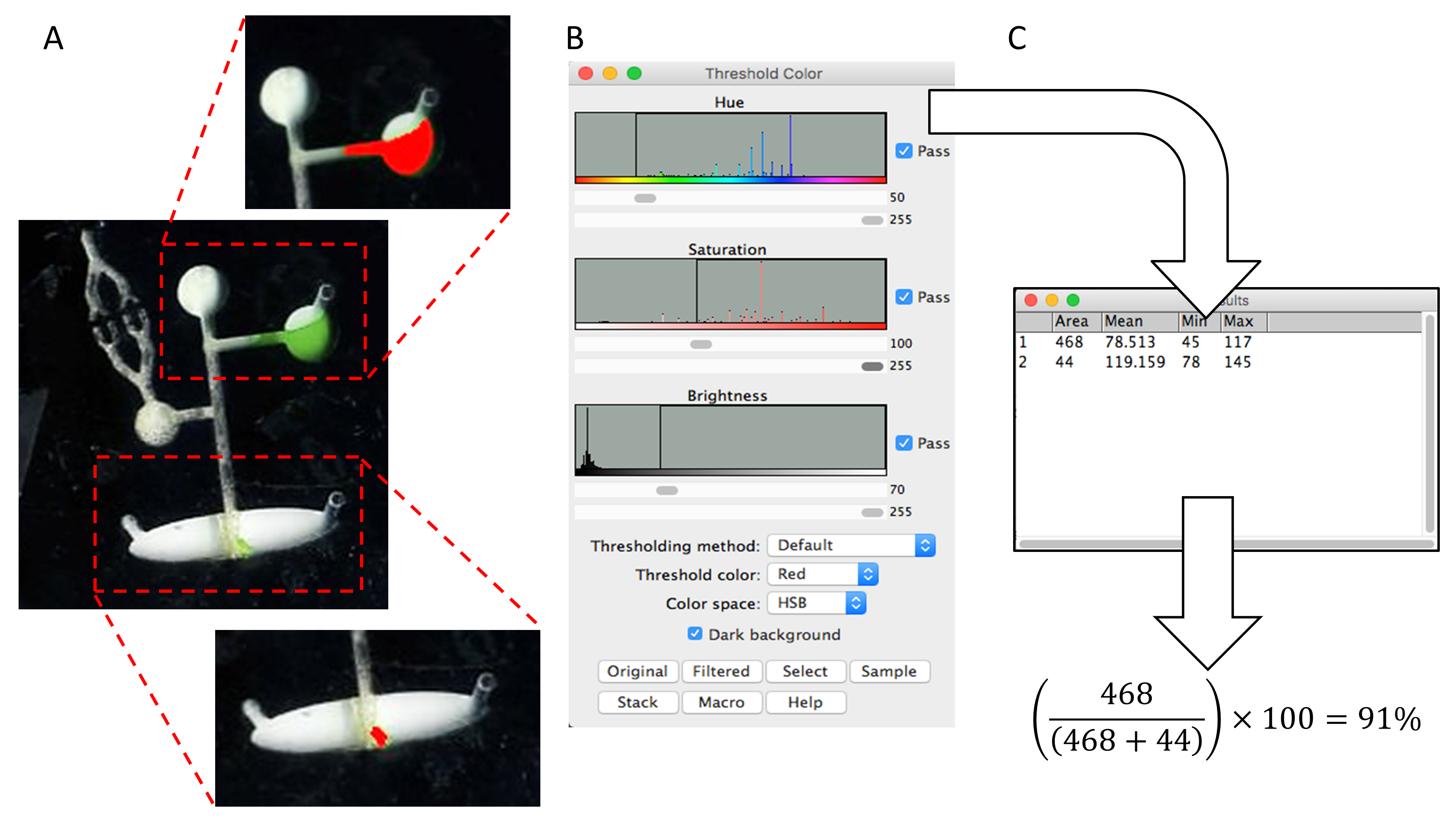

Supplement: Supplementary file 1 [file micromachines-11-00627-s001.zip › Slide2.TIF]
